# Knowledge, attitudes, and practices of primary care general practitioners regarding COPD inhaler education: a cross-sectional study in Yiwu, China

**DOI:** 10.3389/fphar.2026.1795439

**Published:** 2026-07-14

**Authors:** Dingjin Lou, Yiming Teng, Jingjing Ren, Zhening Yang, Mingmin Chen

**Affiliations:** 1 General Practice Department, Yiwu Central Hospital, Yiwu, China; 2 General Practice Department, The First Affiliated Hospital, Zhejiang University School of Medicine, Hangzhou, China

**Keywords:** challenges, chronic obstructive pulmonary disease, general practice, inhalation, inhaled therapy, management of COPD, primary healthcare

## Abstract

**Objectives:**

To understand GPs’ (General Practitioners’) knowledge, attitudes, and practices concerning inhalation education for COPD patients; to pinpoint key discrepancies between knowledge, attitude, and behavior; to offer evidence for designing multi-level targeted training and implementation strategies that can enhance the quality of inhaler education provided at the grassroots level.

**Methods:**

A cross-sectional, web-based study was conducted among GPs in Yiwu, Zhejiang Province, China, from November to December 2025. A validated, self-administered questionnaire assessed KAP across three dimensions (knowledge, attitude, practice). Descriptive statistics, Kruskal–Wallis H test, correlation analysis, and multiple regression were used for data analysis.

**Results:**

Among the 213 participating GPs, mean standardized scores (0–100) were 31.2 ± 9.0 for knowledge, 84.1 ± 12.5 for attitude, and 41.0 ± 15.4 for practice. Lower knowledge scores were observed among GPs with junior professional titles (28.4 ± 9.0) and those with less than 5 years of clinical experience (27.1 ± 9.1). Multivariate regression showed that knowledge was independently associated with practice behavior (standardized β = 0.508, P < 0.001).

**Conclusion:**

This study indicates that GPs in Yiwu showed positive attitudes but insufficient knowledge and practice in COPD inhaler education, with a clear attitude-practice gap. These findings may support the development of targeted strategies to improve GPs’ engagement in inhaler technique education for COPD patients.

## 1 Introduction

Chronic obstructive pulmonary disease (COPD) is the third leading cause of death globally, responsible for over 3.23 million deaths annually (Chronic obstructive pulmonary disease, 2025; Alupo et al., 2024). Moreover, previous data demonstrated that COPD diseases are aggressively growing in absolute numbers (Gbd, 2020). Recent findings from a collaborative study project that the global economic burden of COPD will amount to INT$4·326 trillion (with a range of 3·327–5·516 trillion at constant 2017 prices) in 2020-2050,and China face the largest economic burdens, accounting for INT$1·363 trillion (uncertainty interval 1·034–1·801), without practical and effective improvements, the global economic burden of COPD could grow enormously in the coming decades (Chen et al., 2023). The anticipated increase in COPD cases underscores the pressing need to tackle the challenges associated with effective management and prevention to reverse this trend. The 2025 GOLD report further emphasizes the importance of inhalation therapy as a cornerstone of COPD management, due to its ability to deliver bronchodilators and anti-inflammatory agents directly to the lungs, resulting in rapid and identifiable clinical effects. International guidelines, including those of the GOLD, uniformly recommend that inhalers be prescribed and their use regularly reviewed (Alupo et al., 2024).

Identified clinical benefits of inhalers are critically dependent on the persistent use of correct technique, as detailed in various studies and guides (Alcázar-Navarrete et al., 2022; Kocks et al., 2023; Leving et al., 2022; Nordon et al., 2025). Studies have documented that improper inhaler technique is common among patients, leading to inadequate symptom management, more frequent exacerbations, and increased healthcare utilization and mortality (Jian, 2013; Izquierdo-Condoy et al., 2024; Willard-Grace et al., 2020; Marko and Pawliczak, 2025). Strikingly, previous research performed in multi-countries exhibited over 80% of the instances where patients made errors in using inhalation devices (Kocks et al., 2023). We hypothesize that among community patients, ineffective and irregular inhaler use is likely more prevalent. Consequently, primary healthcare providers are urged to repeatedly educate, assess and remedy patients at every contact (Marko and Pawliczak, 2025). This approach would represent a cost-effective strategy to address the challenge posed by the COPD disease burden (Shaikh et al., 2025).

General practitioners (GPs) have a significant responsibility in managing COPD patients, including patient education, treatment during the stable phase, and long-term follow-up. What’s more, GPs working at primary-care facilities are the first—and sometimes the only—point of contact for most COPD patients in China. They are expected to select the appropriate device, provide hands-on training, and perform follow-up evaluations. Nevertheless, a number of previous studies showed that even medical workers lack sufficient knowledge and practice skills on inhaler technique (Kang et al., 2021; Fink and Rubin, 2005; Qiaohui, 2024; Xie et al., 2023), not to mention instructing and monitoring COPD patients for proper use of them. Furthermore, some researchers suspected things might be even worse in primary healthcare providers (Hanania et al., 1994). In addition, with various newer inhalers progressively emerging, primary caregivers may face further increasing challenges nowadays. However, despite substantial evidence indicating suboptimal knowledge and practice skills among patients and medical workers regarding inhaler techniques, inadequate and impractical strategies have been implemented to address this challenge.

The Knowledge, Attitude, and Practice (KAP) framework is a widely used behavioral model in public health and clinical research that conceptualizes health-related behaviors as being shaped by three interrelated dimensions (Andrade et al., 2020).

Knowledge: Factual understanding, cognitive awareness, and technical proficiency regarding a specific health intervention or practice.

Attitude: Personal beliefs, perceived importance, willingness, and motivational orientation toward the intervention.

Practice: Actual behavioral performance, implementation, and adherence to the recommended clinical actions in routine care.

The KAP model is particularly suitable for investigating barriers to effective inhaler education among GPs, because it systematically disentangles what they know, what they believe, and what they actually do—a critical distinction for identifying barriers to consistent, high-quality inhaler education. It enables a quantitative assessment of gaps between cognitive, motivational, and behavioral domains, which is essential for designing targeted interventions.

To our best knowledge, comprehensive, region-specific KAP data on primary-care GPs are lacking in eastern China. To address this gap, we conducted a cross-sectional, web-based study involving 213 GPs practicing in healthcare settings of varying levels across Yiwu. Yiwu has 1.92 million permanent residents, with healthcare services provided by 47 hospitals, 14 community health centers (including township health centers), 179 village clinics, and 60 community health service stations (2024 Statistical Bulletin on the National Economy and Social Development of Yiwu City, 2025). As a typical county-level city in eastern China with a booming commodity economy, Yiwu’s primary healthcare system shares core characteristics with other urbanized regions in China: (i) a three-tiered service network (hospitals→community/township health centers → community health service stations/village clinics) covering urban and rural areas; (ii) GPs as the first point of contact for chronic disease management, responsible for diagnosis, treatment, education, and long-term follow-up of COPD patients; (iii) consistent national guidelines for COPD management (aligned with the GOLD report) and standardized training requirements for GPs. These features are relatively common in urbanized county-level regions in eastern China; therefore, the findings regarding GPs’ knowledge gaps and practice barriers may provide a useful reference for primary care settings with similar socioeconomic and healthcare contexts.

The objectives of our study were: (i) to understand knowledge, attitudes, and practices concerning inhaler technique education for COPD patients; (ii) to pinpoint key discrepancies between knowledge, attitude, and behavior; and (iii) to offer evidence for designing multi-level and multifaceted targeted training and implementation strategies that can enhance the quality of inhaler education provided at the grassroots level.

## 2 Methods

### 2.1 Study design and setting

We conducted a cross-sectional, web-based study in Yiwu, a city in Zhejiang Province, China. The fieldwork was conducted from 25 November to 15 December 2025.

### 2.2 Participants

Inclusion criteria: (i) GPs practicing in Yiwu; (ii) those involving managing COPD patients; (iii) individuals willing to complete an anonymous questionnaire. Exclusion criteria: respiratory specialists, nurses, or doctors not involved in direct patient care. The minimum sample size was calculated based on the rule of thumb for questionnaire studies, requiring 5–10 participants per item. The questionnaire contained 21 items, yielding a target sample size of 105–210. Sample size was further verified using G*Power 3.1 software and the required sample size was 74. Finally, a total of 243 questionnaires were collected, among which 213 were valid (validity rate: 87.7%), which was sufficient for statistical analysis.

### 2.3 Questionnaire

The initial version of the self - administered questionnaire was designed based on literature review. Subsequently, expert consultation was performed with one specialist in general practice and two specialists in respiratory medicine. The questionnaire was then refined via two rounds of revision in accordance with experts feedback, and additional items were incorporated into the practice section to improve comprehensiveness. Finally, a pilot investigation was conducted among 20 GPs before the formal investigation to assess the clarity, understandability, rationality, and applicability of the questionnaire items.

The questionnaire comprised two sections. Section A focused on background information, including age, gender, institution level, clinical experience, professional title, and education level. Section B was a 21-item KAP instrument, including 10 knowledge items, five attitude items, and six practice items. Items 1–20 were five - point scales (1–5) indicating the degree of knowledge, agreement and practice performance (i.e., “extensive knowledge” was scored five points, “good knowledge” 4 points, “moderate knowledge” 3 points, “minimal knowledge” two points, and “no knowledge at all” one point). Item 21 was a question with multiple choice, specifically, the methods employed to assist inhaler instruction, each method was one point, totaling five points. Thus, the total score of these three dimensions was 50 points, 25 points and 30 points, respectively. Following the manual scoring for each dimension, the average points were calculated, obtaining the “raw scores”, and a linear transformation was applied to obtain a 0–100 value. High values reflected a superior knowledge, attitude or behavior. More details about the questionnaire were presented in the Supplementary Material.

The Cronbach’s α coefficient was calculated to evaluate the internal consistency reliability of the questionnaire. In this study, the Cronbach’s α was 0.932, indicating excellent reliability of the questionnaire. Exploratory factor analysis with principal component extraction and varimax rotation was performed to evaluate construct validity. All factors yielded eigenvalues > 1, and all items presented factor loadings ≥ 0.5, which indicated good construct validity.

### 2.4 Procedures

An invitation containing a brief description and a unique Quick Response (QR) code linking to the Wenjuanxing® platform was distributed through: (i) institutional WeChat work-groups; (ii) face-to-face contact by township coordinators. Electronic informed consent was obtained on the opening page.

### 2.5 Data analysis

Data were exported to SPSS 26.0. Incomplete questionnaires (<80% answered) were excluded. Qualitative data were summarized using absolute and relative frequencies, and quantitative data were reported in terms of mean and SD. As the data were ordinal Likert-scale measurements, nonparametric tests were used for group comparisons. The Kruskal–Wallis H test was applied to compare differences across multiple subgroups. Normality, homogeneity of variance, and residual diagnostics were not performed because nonparametric methods do not require these assumptions. Spearman’s rank correlation was utilized to evaluate the K–A, K–P, and A–P relationships. Multiple regression analysis was carried out to identify the influential factors affecting GPs’ practice in inhalers instruction. A two-sided p-value less than 0.05 was deemed statistically significant.

## 3 Results

### 3.1 Characteristics of participants and overall KAP scores

A total of 213 valid GPs were included in the analysis. The mean age was 35.7 ± 11.7 years. There were 109 males (51.2%) and 104 females (48.8%). Most participants had less than 5 years of clinical experience (89, 41.78%), and held junior professional titles (114, 53.52%). For institution type, 40 (18.78%) worked in general hospitals, 95 (44.60%) in community healthcare centers, 44 (20.66%) in township health centers, 17 (7.98%) in village health clinics, and 17 (7.98%) in community healthcare stations. Regarding education level, 10 participants (4.69%) held a postgraduate or higher degree, 175 (82.16%) held a bachelor’s degree, and 28 (13.15%) had education below bachelor’s degree (Table 1).

**TABLE 1:** Characteristics of GPs and comparison of KAP regarding inhaler technique education (N = 213).

Item	No.	%	Knowledge	Attitude	Practice
Institution type	​	​	31.2 ± 9.0	84.1 ± 12.5	41.0 ± 15.4
General hospital	40	18.78	28.0 ± 10.3	84.4 ± 11.3	41.9 ± 17.9
Community health service center	95	44.60	30.9 ± 8.9	84.2 ± 13.3	39.6 ± 15.3
Township health center	44	20.66	33.5 ± 8.8	85.7 ± 11.8	41.0 ± 12.0
Village clinic	17	7.98	29.9 ± 6.8	74.6 ± 9.4	44.3 ± 15.8
Community health service station	17	7.98	35.6 ± 6.1	88.5 ± 11.5	43.7 ± 18.3
P	​	​	0.085	0.002	0.974
Sex	​	​	​	​	​
Male	109	51.20	31.3 ± 8.7	83.5 ± 12.2	42.9 ± 14.8
Female	104	48.80	31.1 ± 9.4	84.8 ± 12.8	39.0 ± 15.8
P	​	​	0.444	0.392	0.003
Age range,y	​	​	​	​	​
<30	83	38.97	26.7 ± 8.9	82.7 ± 11.3	39.0 ± 13.5
30∼39	56	26.29	34.8 ± 8.9	87.8 ± 14.2	42.8 ± 17.0
40∼49	43	20.19	33.3 ± 7.4	83.9 ± 12.1	41.9 ± 17.0
50∼59	29	13.62	34.1 ± 6.6	81.9 ± 11.7	42.3 ± 15.8
Lost	2	3.30	​	​	​
P	​	​	0.073	0.198	0.514
Duration of work experience (years)	​	​	​	​	​
<5	89	41.78	27.1 ± 9.1	83.3 ± 11.2	39.0 ± 14.1
5∼10	36	16.90	34.6 ± 8.4	87.3 ± 16.4	43.8 ± 17.4
11∼20	45	21.13	34.0 ± 8.9	87.1 ± 10.4	42.2 ± 15.3
>20	43	20.19	33.8 ± 5.8	80.1 ± 12.0	41.6 ± 16.4
P	​	​	0.003	0.001	0.787
Professional title	​	​	​	​	​
Junior	114	53.52	28.4 ± 9.0	82.5 ± 13.3	40.4 ± 14.8
Intermediate	65	30.52	33.8 ± 8.1	86.8 ± 10.2	41.9 ± 15.7
Associate senior	24	11.27	34.7 ± 7.1	82.7 ± 14.2	41.1 ± 18.0
Senior	10	4.69	37.0 ± 9.6	88.8 ± 9.0	42.7 ± 15.8
P	​	​	0.001	0.109	0.896
Education	​	​	​	​	​
Postgraduate degree or above	10	4.69	31.0 ± 10.2	82.8 ± 11.5	39.3 ± 15.1
Bachelor	175	82.16	30.9 ± 9.3	84.8 ± 12.4	40.9 ± 15.1
Below	28	13.15	32.9 ± 6.5	80.6 ± 13.1	42.6 ± 18.0
P	​	​	0.71	0.069	0.814

Test: Kruskal–Wallis H test (nonparametric).

The overall mean scores were 31.2 ± 9.0 for knowledge, 84.1 ± 12.5 for attitude, and 41.0 ± 15.4 for practice, respectively. Participants demonstrated highly positive attitudes but insufficient knowledge and suboptimal practice regarding inhaler technique education for COPD patients. Statistically significant differences in attitude scores were observed across different institution types (P < 0.05), while no notable variations were found in knowledge and practice. Additionally, knowledge, attitude and practice showed no significant differences among participants with varying education levels (all P > 0.05) (Table 1).

### 3.2 Knowledge dimension

**FIGURE 1:**
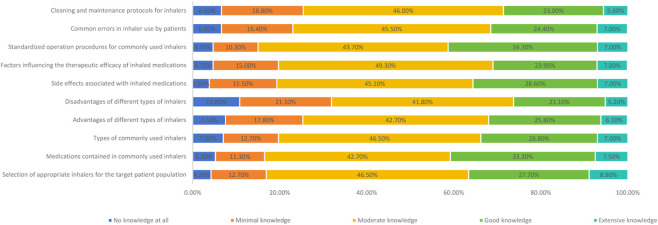
Knowledge towards inhaler technique education (N = 213).

The mean knowledge score was 31.2 ± 9.0 out of 100, indicating insufficient knowledge. Most GPs (41.80%–49.30%) selected “moderate knowledge” for each item. Relatively better knowledge was reported for drug components of common inhalers (33.30% good, 7.50% extensive) and standardized operation procedures (34.30% good, 7.0% extensive). Figure 1 shows the proportion for each answer. Knowledge scores differed significantly by professional title and clinical experience (P < 0.05). GPs with junior title had a lower score (28.4 ± 9.0), and those with <5 years of experience also scored lower (27.1 ± 9.1). No significant differences were found by gender, institution type, age, or education level (P ≥ 0.05) (Table 1).

### 3.3 Attitude dimension

**FIGURE 2:**
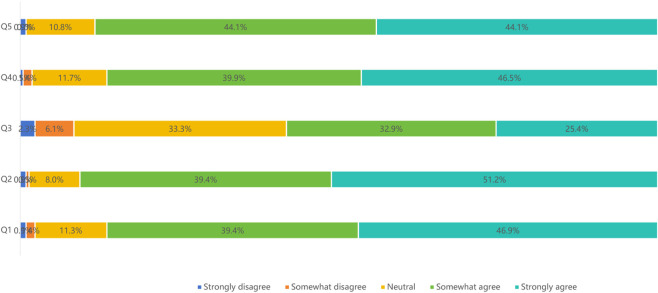
Attitude towards inhaler technique education (N = 213).

The mean attitude score was 84.1 ± 12.5 out of 100, reflecting strongly positive attitudes. Over 85% of GPs agreed that correct inhaler selection and technique are crucial for COPD outcomes. However, only 25.4% strongly agreed that they provided standardized instruction, while 32.9% somewhat agreed. In addition, GPs showed a strong desire to receive professional training on this aspect (39.9% somewhat agreed, 46.5% strongly agreed) and to obtain more education tools (44.1% somewhat agreed, 44.1% strongly agreed). Figure 2 presents agreement rates for each item. Attitude scores differed significantly by institution type and clinical experience (P < 0.05). GPs in village clinic had a lower score (74.6 ± 9.4), and those with >20 years of experience also scored lower (80.1 ± 12.0). No significant differences in attitude scores were observed across age, gender, professional title, or education level (P ≥ 0.05) (Table 1).

Q1: Do you agree that tailoring individualized inhalers is crucial for clinical outcome of COPD patients?
Q2: Do you agree that correct use of inhalers is crucial for clinical outcome of COPD patients?
Q3: Do you agree that you provide standardized instruction on inhaler technique for COPD patients?
Q4: Do you wish to receive professional training about inhaler devices?
Q5: Do you wish to obtain more auxiliary tools assisting inhaler device instruction (e.g., placebo devices, brochures, videos)?

### 3.4 Practice dimension

The mean practice score was 41.0 ± 15.4 out of 100, indicating inadequate implementation of inhaler education. Over the past month, 55.4% of GPs reported instructing only 1-5 COPD patients per week. For one-to-one demonstrations with first-time users, 35.2% of GPs spent ≤3 min, and 35.2% spent 4–6 min. Regarding the proportion of patients rechecked after instruction, 50.2% of GPs reported less than a quarter. 39% of GPs had never heard of the “standard 7-step checklist”, while only 23% of GPs occasionally use it to assess patient mastery. Moreover, most GPs (55.9%) have never documented inhaler technique assessment in patient records. Generally, GPs instructed patients on how to use inhalers through verbal explanation (80.3%) and physical demonstration (63.4%). More detials were showed in Table 2. Practice scores differed significantly by gender, with female participants recording lower values (39.0 ± 15.8, P < 0.05). No significant differences were detected among subgroups based on age, clinical experience, institution type, professional title, or education level (P ≥ 0.05) (Table 1).

**TABLE 2:** Practice regarding inhaler technique education (N = 213).

Item	Response option	n	%
1. Average number of patients you instructed them on inhaler technique per week (past month)	0	63	29.6
	1 ∼ 5	118	55.4
	6 ∼ 10	23	10.8
	11-20	6	2.8
	>20	3	1.4
2. Time spent on one-to-one demonstration for first-time users	Never demonstrated	47	22.1
	≤3min	75	35.2
	4 ∼ 6min	75	35.2
	7 ∼ 10min	13	6.1
	>10min	3	1.4
3. Proportion of patients rechecked after instruction	<25%	107	50.2
	25 ∼ 49%	49	23
	50 ∼ 74%	31	14.6
	75 ∼ 99%	20	9.4
	100%	6	2.8
4. Use of a “standard 7-step checklist” to assess patient mastery	Never heard of it	83	39
	Heard of it but never used	57	26.8
	Occasionally	49	23
	Frequently	21	9.9
	Every time	3	1.4
5. Documentation of inhaler technique assessment in medical records	Never	119	55.9
	<30%	48	22.5
	30 ∼ 59%	23	10.8
	60 ∼ 89%	18	8.5
	>90%	5	2.3
6. Teaching methods employed (multiple responses allowed)	Verbal explanation	171	80.3
	Physical demonstration	135	63.4
	Teach-back by patient	69	32.4
	Video /leaflet	66	31
	Family member training	53	24.9

### 3.5 Knowledge–attitude–practice correlations and multiple regression analysis

Spearman correlation showed significant associations: knowledge–attitude (r = 0.277), knowledge–practice (r = 0.504), and attitude–practice (r = 0.188; all P < 0.001). Multiple regression revealed that knowledge was independently associated with practice (standardized β = 0.508, P < 0.001). No multicollinearity was observed (VIF < 2) (Table 3).

**TABLE 3:** Multiple regression analysis for GPs practice in inhalation education for COPD patients.

​	Standardization coefficient	t	P	Beta 95% IC		Collinearity statistics	
—	Beta	—	—	Lower limit	Upper limit	Tolerance	VIF
(Constant)	—	1.499	0.135	−0.999	7.338	—	—
Knowledge	0.508	7.853	0	0.392	0.654	0.849	1.178
Attitude	0.053	0.852	0.395	−0.104	0.263	0.911	1.098

## 4 Discussion

### 4.1 Principal findings

This cross-sectional study of 213 primary care general practitioners in Yiwu, China, revealed substantial deficits in knowledge regarding inhaler technique education for COPD patients, along with a pronounced attitude–practice gap despite highly positive attitudes. Multivariate regression indicated that knowledge was independently associated with inhaler education practice, whereas attitude exhibited no independent predictive effect. Compared with previous KAP studies in China and internationally, the present study is unique in quantitatively confirming the attitude–practice gap and identifying knowledge was independently associated with teaching behavior. These findings clarify the key barrier to high-quality inhaler education and provide evidence for multi-level strategies for improving primary care COPD management.

Our study showed that GPs demonstrated substantial insufficiency in knowledge related to inhaler devices and patient education. These results are consistent with studies conducted in China, which have repeatedly confirmed that primary care providers lack adequate theoretical knowledge about inhaler use (Kang et al., 2021). Yiwu’s experience resonates with primary care contexts in countries with rapidly aging populations and increasing COPD burdens (e.g., India, Brazil, and parts of Europe). Studies in these regions have also reported that primary care providers lack knowledge of inhaler technique, despite recognizing its clinical importance (Alupo et al., 2024; Papaioannou et al., 2025). Poor knowledge likely stems from limited formal training during medical education and insufficient continuing education updates, especially as new inhaler devices continue to emerge. Furthermore, the time constraints, limited educational tools, and lack of performance incentives faced by Yiwu’s GPs are universal barriers reported in primary care settings globally (O'Dwyer et al., 2020; Willard-Grace et al., 2020). These parallels indicate that our findings are not confined to Yiwu but reflect broader systemic challenges in primary care COPD management, enhancing the external validity of our research.

Although GPs held strongly positive attitudes toward the importance of standardized inhaler education, their actual teaching performance remained suboptimal. Our results are consistent with previous studies indicating that approximately one - third of COPD patients have never received education on how to use inhalers properly (Dabrowska et al., 2019), although evidence confirms that errors made by patients decreased significantly when they received education (Marko and Pawliczak, 2025). Attitude - practice gaps have been widely documented, aligning with the “attitudinal–behavioral split” described in the Capability - Opportunity - Motivation - Behavior (COM-B) framework (Michie et al., 2011), which posits that “capability” (here, knowledge) is a prerequisite for behavior change—even with strong “motivation” (attitude).

Multiple regression analysis identified knowledge was independently associated with clinical practice, while attitude showed no independent effect. This finding strengthens the argument that improving professional knowledge is essential to enhancing inhaler education quality. Previous domestic study have similarly demonstrated that knowledge level is strongly associated with healthcare providers’ practice, while attitude exhibits no significant mediating effect between knowledge and practice (Jiaming et al., 2024). Internationally, this result is supported by studies across several health systems, which conclude that targeted knowledge training is the key to closing implementation gaps (Alupo et al., 2024). These consistent observations highlight that interventions must prioritize competency-based knowledge training. However, Al-Hamaden et al. found that healthcare professionals involved in asthma care possessed sufficient knowledge yet showed poor adherence to clinical guidelines (Al-Hamaden et al., 2024). This suggests that continuing education and training alone are not enough. Integrating clinical guidelines into institutional standard operating procedures and carrying out regular performance audits may therefore be essential.

## 5 Implications for policy and practice

Our findings support the urgent implementation of multi-level interventions. Policymakers could incorporate inhaler education quality into primary care performance evaluations and offer incentives to GPs with excellent performance in inhaler education. Healthcare institutions could establish regular competency-based training programs and provide diverse educational tools, such as instructional videos and leaflets. Healthcare institutions could integrate clinical guidelines into institutional standard operating procedures and carry out regular performance audits. Medical schools and continuing education platforms could reinforce inhaler-related content across undergraduate, postgraduate, and lifelong learning curricula. Pharmaceutical industries can assist by developing user-friendly inhaler devices and supplying free demonstration models. Collectively, these strategies can systematically enhance inhaler education capacity in primary care settings.

## 6 Limitation

This study has several limitations. First, the sample was limited to general practitioners in Yiwu, which limits the generalizability of the results. Second, knowledge and practice were measured by self-reported questionnaires rather than objective assessments, and we were unable to observe participants’ actual clinical performance. Third, the online voluntary survey carries risks of self-selection bias and nonresponse bias, given the unknown profiles of non-participants. Fourth, multiple subgroup analyses were conducted without statistical correction, potentially leading to inflated type I error. Fifth, the cross-sectional design only reflects a single time point, so causal relationships cannot be established. Interventional studies are needed to confirm causality. Sixth, we did not record individual GPs’ monthly or weekly volume of COPD patients, leaving the impact of clinical workload on knowledge and practice unexamined. Future studies should incorporate this indicator. Seventh, adherence to GOLD guidelines for inhaler education was not assessed in this research, and relevant evaluation is suggested for future work to improve clinical relevance.

## 7 Conclusion

Our study indicates that primary care GPs in Yiwu had positive attitudes but insufficient knowledge and practice regarding COPD inhaler education, with a clear attitude-practice gap. Knowledge was independently associated with teaching practice. These findings may support the development of targeted strategies to improve GPs’ engagement in inhaler technique education for COPD patients.

## Data Availability

The original contributions presented in the study are included in the article/Supplementary Material, further inquiries can be directed to the corresponding author.
